# Impulsivity in Bipolar Disorder: State or Trait?

**DOI:** 10.3390/brainsci12101351

**Published:** 2022-10-05

**Authors:** Rachel Primo Santana, Jess Kerr-Gaffney, Anda Ancane, Allan H. Young

**Affiliations:** 1 Department of Psychological Medicine, Institute of Psychiatry, Psychology and Neuroscience, King’s College London, London SE5 8AB, UK; 2 South London and Maudsley NHS Foundation Trust, London SE5 8AZ, UK

**Keywords:** bipolar disorder, euthymia, impulsivity, inattention, response inhibition, delayed gratification

## Abstract

Impulsive behaviour is a key characteristic of mania in bipolar disorder (BD). However, there is mixed evidence as to whether impulsivity is a trait feature of the disorder, present in the euthymic state in the absence of mania. The aim of this systematic review and meta-analysis was to examine whether impulsivity is elevated in euthymic BD in comparison to controls. Electronic databases were searched for papers published until April 2022 reporting data on a self-report or behavioural measure of impulsivity in a euthymic BD group and a healthy control group. In total, 46 studies were identified. Euthymic BD showed significantly higher levels of self-reported impulsivity compared to controls (large effect size). Euthymic BD also showed significantly higher levels of impulsivity on response inhibition and inattention tasks, with moderate and large effect sizes, respectively. Only two studies measured delay of gratification, finding no significant differences between groups. Our results suggest impulsivity may be a trait feature of BD, however longitudinal cohort studies are required to confirm whether elevated impulsivity is present before illness onset. Future research should establish whether cognitive interventions are beneficial in improving impulsivity in BD.

## 1. Introduction

Impulsivity is a symptom of mania in bipolar disorder (BD), reflecting a tendency towards fast and unplanned responses with disregard of the consequences for oneself or others [1]. Importantly, impulsivity is associated with greater functional impairment, higher rates of hospitalisations, higher suicide risk, and illness severity in BD [2,3,4]. While several studies have shown higher rates of impulsivity on self-report and behavioural measures of impulsivity for mania in BD [5,6], findings are mixed when measuring impulsivity in euthymic patients. For example, while some studies have found no differences between remitted BD patients and controls across different measures of impulsivity [7], others have reported higher levels of impulsivity in all phases of illness: mania, depression, and euthymia [6]. Furthermore, some studies have found elevated impulsivity in unaffected relatives of those with BD, compared to controls [8,9]. This has led to the suggestion that impulsivity may be a trait feature of BD, present before and after the acute phase of illness [10]. Clarifying the nature of impulsivity in BD may help the development of more targeted treatment plans and consequently improve illness burden among people with BD.

The discrepant findings regarding impulsivity in euthymic BD are likely due in part to the different ways in which impulsivity has been measured. There is currently no consensus on how best to measure impulsivity, however the Barratt Impulsiveness Scale (BIS) is perhaps most common in BD research. The BIS is a self-report scale based on Patton et al.’s [11] model of impulsivity, encompassing three aspects of impulsivity: attentional, motor, and non-planning. Attentional impulsivity refers to the inability to focus on tasks, or distractibility. Motor impulsivity refers to the inability to inhibit responses when required. Finally, non-planning impulsivity refers to the tendency to choose immediate rewards over better ones in the future (delay of gratification). Some studies have suggested that certain aspects of impulsivity, measured via the BIS, are more strongly related to certain mood episodes in BD. For example, Swann et al. [12] found that mania was associated with motor impulsivity, whereas depression was associated with non-planning impulsivity. Among manic symptoms, visible hyperactivity was most strongly correlated with BIS scores, whilst anhedonia, hopelessness, and suicidality were the depressive symptoms most strongly correlated with BIS scores. Thus, different aspects of impulsivity may be differentially related to mood states in BD.

There are important limitations to self-report measures of impulsivity. In studies with euthymic participants, it is possible that respondents think back to instances where they have been impulsive during mania or subsyndromal periods, rather than when they have been euthymic. This recall bias would therefore inflate self-report impulsivity scores. Consequently, some studies have employed behavioural paradigms to measure various aspects of impulsivity in individuals with BD. Response inhibition, or motor impulsivity, has perhaps received most attention. A common measure of response inhibition is the stop signal task (SST), whereby individuals are instructed to press a button quickly and accurately when they see a specific target. However, in some trials a stop signal is presented before the stimulus, instructing the respondent to inhibit their response. Similar tasks include the Hayling Sentence Completion Task (HSCT), go/no-go task, the Continuous Performance Test (CPT), and the Immediate Memory Task (IMT). Results from studies using these tasks are mixed, with some finding an increased impulsivity in euthymic BD compared to controls [13] while others have found no differences [7,14]. Methodological differences across studies are likely to have impacted these findings. For example, Van der Shoot et al. [15] reported that auditory stop signals in the SST produce faster and more accurate responses than visual stop signals. Similarly, participant characteristics, such as age and gender, have also been found to influence performance on response inhibition tasks [16,17].

Other behavioural studies have measured inattention, another component of impulsivity. Most studies in BD have used the CPT, in which participants must continually press a button for a series of letters as they appear on the screen, however, they need to withhold their response when the letter X appears. While commission errors (responding to the X when withholding a response is required) are taken as a measure of response inhibition, omission errors (failing to respond to letters other than X) are taken as a measure of inattention. Studies in manic participants have found a worse performance on the CPT when compared with controls [18], and some have also reported a worse performance in remitted BD patients [4,19]. These studies also reported that a poorer performance was related to more past hypomanic episodes and hospital admissions, suggesting that severity or duration of illness may have an impact on this aspect of impulsivity. Similarly, attentional impulsivity as measured by the BIS has been found to predict severity of illness one year later [3]. Given that inattention is present in both manic and euthymic phases of BD, it is possible that this is a trait feature of BD. However, since there are also variations with illness severity and possibly duration, inattention may be a result of prolonged illness, similar to other cognitive impairments associated with BD [20].

Finally, delay of gratification (non-planning impulsivity in Patton et al.’s [11] model) has also been examined in BD. Delay of gratification tasks include the delay discounting task, Cambridge Gambling Task (CGT), Single Key Impulsivity Paradigm (SKIP), and the delayed reward task. More impulsive responses are reported in the manic phase of BD compared to controls [18]. However, several studies have found no differences between euthymic patients and controls on delayed gratification tasks [21,22]. Indeed, a longitudinal study reported that while manic participants performed worse on the delayed reward task than controls, this was no longer true when they achieved remission or switched to depression one year later [6]. Overall, it appears that delay of gratification may be related to mania rather than a trait feature of BD, however there are relatively few studies in this area, preventing firm conclusions from being made.

To our knowledge, four systematic reviews examining impulsivity in BD have been published. However, these have either not included a meta-analysis [23,24], included BD groups in any phase of illness [10,23], or restricted their search to either self-report [25] or behavioural studies only [10]. Therefore, the aim of this systematic review and meta-analysis was to investigate whether impulsivity is elevated in euthymic BD compared to healthy controls, potentially reflecting a trait-like feature of the disorder. We aimed to synthesise findings from self-report and behavioural measures separately, allowing effect sizes from studies using different measurement tools to be compared. We also aimed to perform separate meta-analyses for different aspects of impulsivity (inattention, response inhibition, and delay of gratification), to determine whether these may be differentially related to illness state in BD. We hypothesised that both self-reported impulsivity, response inhibition, and inattention would be higher in euthymic BD compared to controls. Due to relatively few previous studies with inconsistent findings, we did not make any predictions regarding delay of gratification.

## 2. Materials and Methods

### 2.1. Protocol

This systematic review and meta-analysis was conducted according to the Preferred Reporting Items for Systematic Reviews and Meta-Analyses (PRISMA) guidelines [26], and pre-registered on PROSPERO (CRD42022311314). A change was made to the original protocol: we used the Kmet [27] quality assessment instead of the Critical Appraisal Skills Programme (CASP) checklist, as it included criteria more relevant to the studies included in this review.

### 2.2. Search Strategy

Three electronic databases (PubMed, PsycInfo, and Web of Science) were searched for primary manuscripts published up until 7 April 2022. All databases were searched using truncation and Boolean operators, using the following search terms: “impulsiv*” OR “delay gratification” OR “inattention” OR “response inhibition” AND “bipolar disorder”. No limits were set on publication date.

### 2.3. Inclusion and Exclusion Criteria

Inclusion criteria for studies in this review were: (a) adult participants (≥18 years); (b) included both a healthy control group and a group with BD; (c) the BD group were euthymic, inter-episode, or in remission, confirmed with a validated measure of mood or diagnostic interview; (d) report data on a measure of impulsivity (self-report or behavioural) in both groups; (e) full text available in English. Exclusion criteria were: (a) studies with less than 10 participants per group, (b) groups with a primary personality disorder diagnosis; (c) systematic reviews, qualitative studies, and case reports.

### 2.4. Study Selection

**Figure 1:**
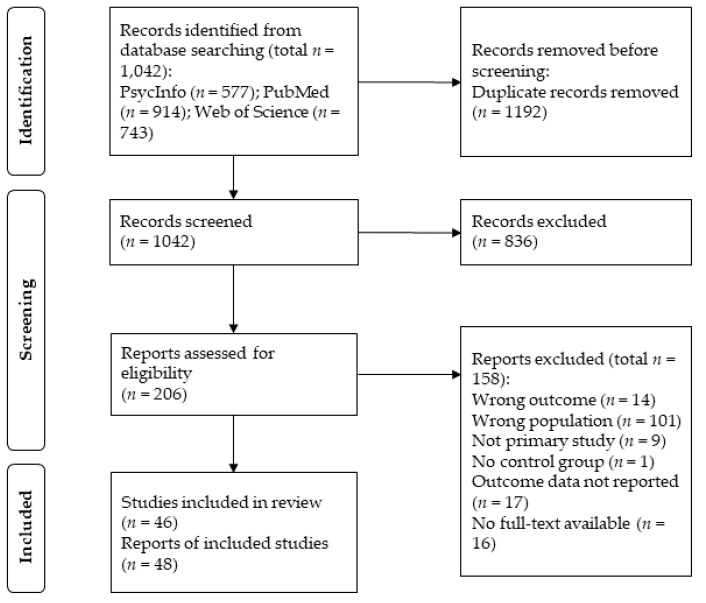
Study selection process.

The study selection process is displayed in Figure 1. In total, the search generated 2234 records. After removing duplicates, records were assessed for relevance based on titles and abstracts. If records were potentially eligible or ambiguous, the record was retained for full-text retrieval. The resulting 206 articles were assessed for eligibility independently by two authors (RPS and JK). Any disagreements on inclusion were settled through a consensus meeting. In total, 48 papers were included, describing 46 studies.

### 2.5. Data Extraction

The following data was extracted from each study: number of participants per group, age, gender (% female), impulsivity paradigm (e.g., stop signal task), impulsivity domain (e.g., response inhibition), and mean and standard deviation (SD) impulsivity scores. Where there was more than one assessment of impulsivity (for example, in longitudinal studies), only the baseline measurement was extracted. Where studies reported variance as standard error, the SD was calculated using the formula SD = SE × √*N*. Where two separate papers described the same sample, these studies were treated as one and data were extracted only once. Study authors were contacted in cases where there was missing or unclear information regarding impulsivity scores.

### 2.6. Study Quality Assessment

A study quality assessment was performed using the Kmet standard quality assessment criteria for evaluating primary research papers [27]. The Kmet form assesses quality of studies on 14 criteria relating to the study design, methods, samples, reporting of results, and conclusions. Three of the criteria did not apply to studies included in this review, as these were related to interventional trial design. The remaining 11 criteria were rated as 0 (no), 1 (partial), or 2 (yes), and summary scores calculated for each study, ranging from 0 to one, with higher scores reflecting higher quality studies. Study quality was assessed independently by two authors (RPS and AA), and any discrepancies resolved by a third author (JK).

### 2.7. Statistical Analysis

Meta-analyses were conducted using Review Manager software (version 5.4.1) [28] and R studio [29] (*metafor* package), using random effects models and standardised mean differences. Separate meta-analyses were carried out for self-report, response inhibition, delay of gratification, and inattention outcome measures. A study could be included in different meta-analyses if it included multiple different measures of impulsivity (e.g., used both a response inhibition task and a delay of gratification task). Cohen’s *d* was used to estimate effect sizes and was reported with 95% confidence intervals (Cis). Effect sizes were interpreted using Cohen’s [30] definitions of small (0.2), medium (0.5), and large (0.8). Positive effect sizes indicated higher impulsivity in the BD group compared to controls. For outcome measures where low scores reflected higher impulsivity, the effect size was reversed before inclusion in the meta-analysis. Between-study heterogeneity was assessed using *I*^2^. This value ranges from 0 to 100%, with higher scores indicating higher heterogeneity. No outlier analysis was performed. Publication bias was assessed through visual inspection of funnel plots and Begg’s rank correlation test [31].

## 3. Results

### 3.1. Study Characteristics

Characteristics of included studies are shown in Table 1 (self-report studies) and Table 2 (behavioural studies). While all studies reported mean age and SD, some studies with a subgroup of euthymic BD only reported the age for the entire BD sample. Mean age ranged from 22.4 to 53.0 years in BD groups, and from 22.5 to 49.9 years in control groups. Three studies used exclusively female samples [32,33,34], however 2 studies [35,36] did not report the sex distribution in at least one group. Thirty-three studies measured euthymia through validated mood measures, 5 used diagnostic measures and 8 studies used both. Twenty-eight studies specified a period of at least 3 to 4 weeks to confirm euthymia, with 5 of these studies recruiting participants that were euthymic for at least 6 months. All, but one study [3], were cross-sectional.

Among the 21 studies measuring response inhibition, 6 used a variation of the CPT, 5 used a form of the go/no-go task, 4 used the SST, 3 the HSCT, 2 the IMT, and 1 the Delis-Kaplan Executive Function System (D-KEFS) colour word interference task. Two studies measured delayed gratification, one study used the SKIP, and another study used the CGT. All five inattention studies used a form of the CPT. All 27 studies using a self-report measure used a version of the BIS.

### 3.2. Quality Assessment

The quality assessment for included studies is presented in Supplementary Table S1. Overall summary scores ranged from 0.55 [52,56] to 0.95 [3,37]. Thirty-three percent of studies scored between 0.85 and 1.0, 56% between 0.70 and 0.84, and 10% between 0.55 and 0.69. Overall, most studies reported variance for the main results, as well as reporting the results in sufficient detail (i.e., both primary and secondary outcomes). Studies often included insufficient sample sizes, and only partially controlled for the confounding factors. The method and reporting of sample selection was also often only partially described.

### 3.3. Synthesis of Findings

#### 3.3.1. Self-Reported Impulsivity

**Figure 2:**
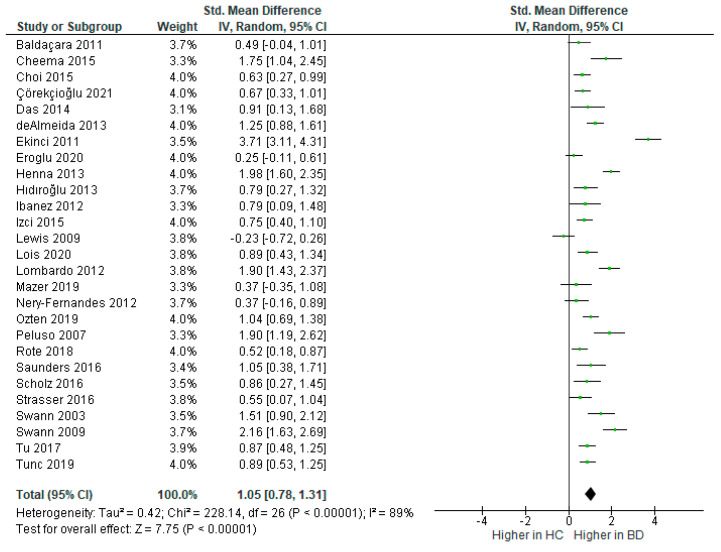
Forest plot of standardised mean differences (SMD) for self-reported impulsivity between bipolar disorder (BD) and healthy controls (HC).

Twenty-seven studies measured self-reported impulsivity. The total number of BD participants was 1269 and controls totalled 1224. The random effects model revealed that impulsivity was significantly higher in BD than controls [*d* = 1.05, 95% CI (0.78, 1.31), *Z* = 7.75, *p* < 0.001], see Figure 2. There was considerable heterogeneity between studies, *I*^2^ = 89%.

#### 3.3.2. Response Inhibition

**Figure 3:**
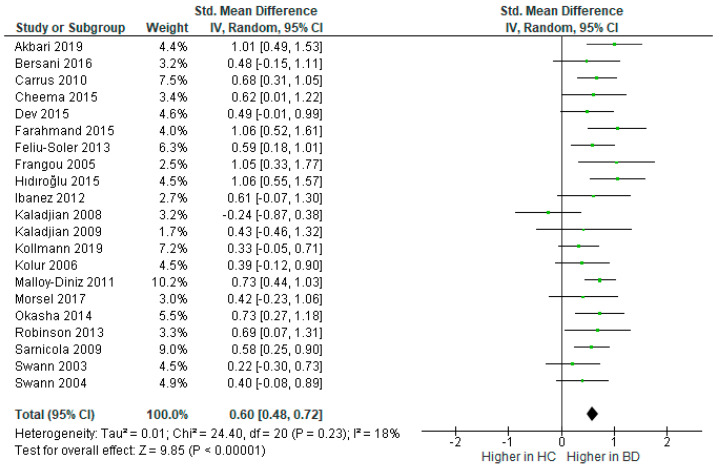
Forest plot of standardised mean differences (SMD) for response inhibition between bipolar disorder (BD) and healthy controls (HC).

Twenty-one studies measured response inhibition. The total number of BD participants was 767 and controls totalled 774. The random effects model revealed that impulsivity was significantly higher in response inhibition tasks in BD compared to controls [*d* = 0.60, 95% CI (0.48, 0.72), *Z* = 9.85, *p* < 0.001], see Figure 3. The between-study heterogeneity was low, *I*^2^ = 18%.

#### 3.3.3. Inattention

**Figure 4:**
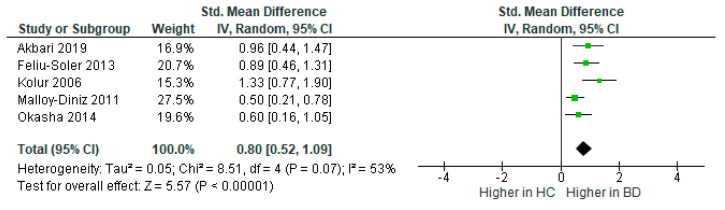
Forest plot of standardised mean differences (SMD) for inattention between bipolar disorder (BD) and healthy controls (HC).

Five studies measured inattention, with 255 BD participants and 254 controls in total. The random effects model revealed that inattention was significantly higher in BD than controls [*d* = 0.80, 95% CI (0.52, 1.09), *Z* = 5.57, *p* < 0.001], see Figure 4. The heterogeneity between studies was moderate, *I*^2^ = 53%.

#### 3.3.4. Delay of Gratification

As there were only two studies measuring delay of gratification, a meta-analysis could not be performed. One study used the CGT [22], while the other used the SKIP [21]. Both studies found no significant differences between euthymic BD participants and controls.

### 3.4. Publication Bias

**Figure 5:**
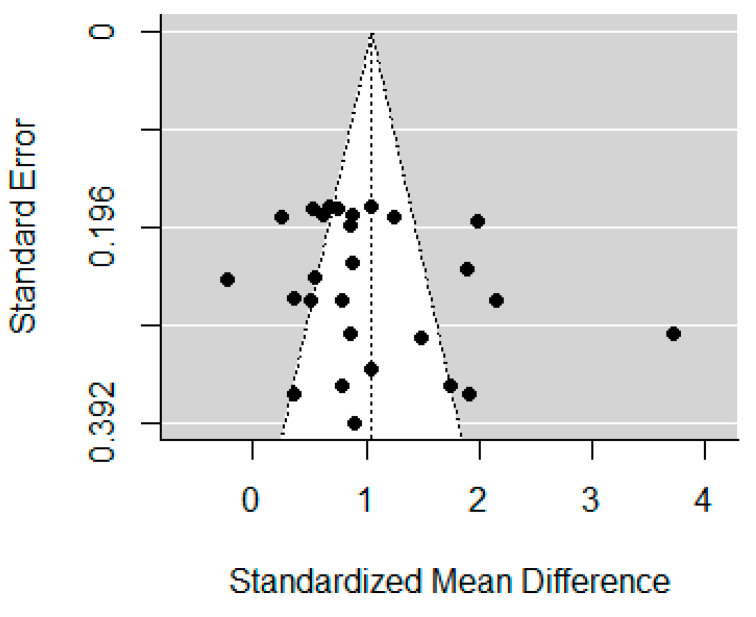
Funnel plot for studies included in the self-report impulsivity meta-analysis.

**Figure 6:**
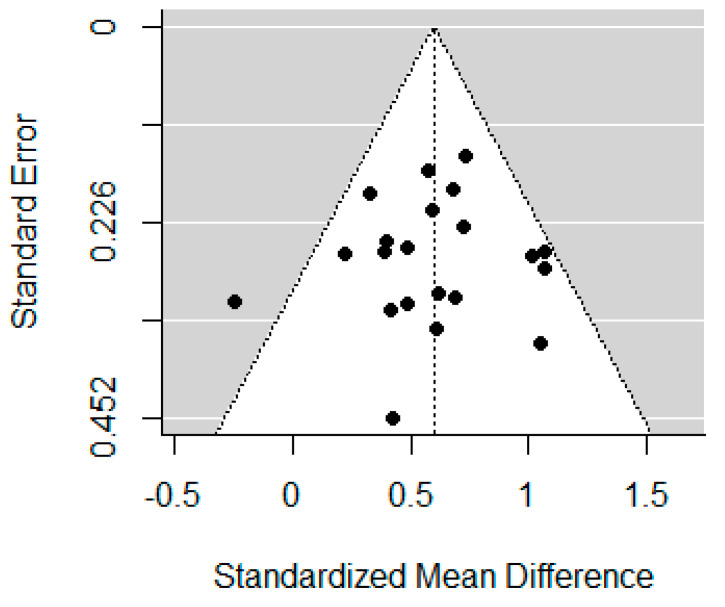
Funnel plot for studies included in the response inhibition meta-analysis.

**Figure 7:**
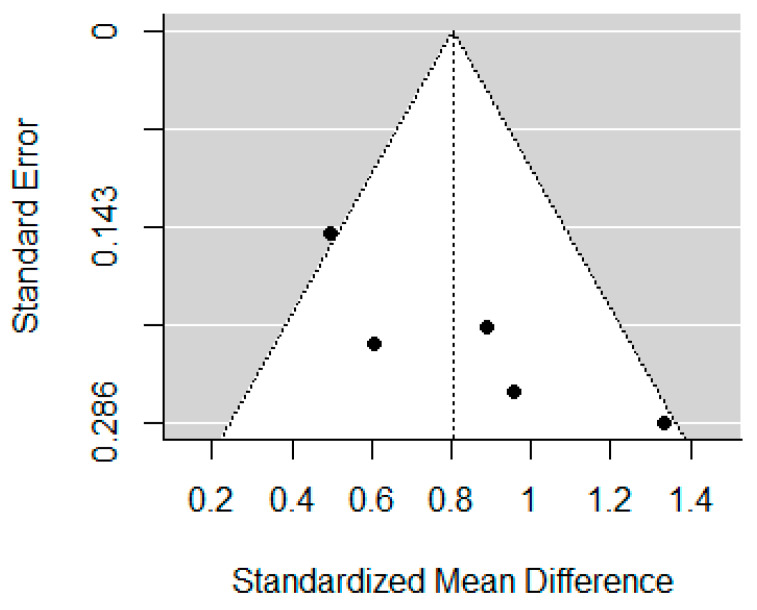
Funnel plot for studies included in the inattention meta-analysis.

The funnel plots for self-report impulsivity, response inhibition, and inattention are displayed in Figure 5, Figure 6 and Figure 7. Studies included in the self-report impulsivity meta-analysis did not show evidence of publication bias (Begg’s test *p* = 0.104), nor did those in the response inhibition meta-analysis (Begg’s test *p* = 0.788). The funnel plot for the inattention studies showed some asymmetry (with a lack of studies in the bottom left corner), however Begg’s test was not significant, *p* = 0.083.

## 4. Discussion

The aim of this systematic review and meta-analysis was to synthesise all available studies using a self-report or behavioural measure of impulsivity in euthymic BD compared to controls. As expected, self-reported impulsivity was significantly higher in BD participants compared to controls, with a large effect size. Considering the results from behavioural studies, euthymic BD participants showed significantly greater impulsivity on tasks measuring response inhibition and inattention, with moderate and large effect sizes, respectively. As only two studies that measured delay of gratification were included, there was insufficient evidence to make conclusions regarding this aspect of impulsivity in euthymic BD. Overall, our findings suggest that impulsivity may be a trait feature of BD, present before and after acute mood episodes.

Our finding regarding higher self-reported impulsivity in euthymic BD compared to controls is consistent with findings from past systematic reviews [23,24], however our study is the first to confirm this in a meta-analysis. We also found high heterogeneity across self-report studies. This could be due to several factors. Firstly, the original BIS was created in the English language, however several of our included studies used a translation of the BIS (e.g., [43,46]). There is limited evidence on the psychometric properties of translated scales, and therefore, different translation techniques could result in the BIS measuring the constructs other than impulsivity in different languages [68]. Secondly, population characteristics such as age and sex may have resulted in heterogeneity between studies. Zelazo and colleagues [16] demonstrated that impulsive behaviour increases during development from childhood to young adulthood, but decreases in older adults. Additionally, specifically in the BD population, studies have shown that males tend to have higher impulsivity scores compared to females [17]. Studies included in the meta-analysis included differing proportions of males and females, and a wide age range of adult BD participants. On the other hand, Ekinci and colleagues [40] reported that BIS scores remained significantly higher in euthymic BD compared to controls, even after controlling for gender, age, and education, suggesting the BIS may be a relatively stable measure of impulsivity.

Impulsivity as measured by response inhibition tasks was also significantly greater in euthymic BD compared to controls, with a moderate effect size. Although the effect size was smaller than for self-report and inattention meta-analyses, the confidence interval was narrower and the heterogeneity between studies much smaller, suggesting more precise estimations. Previous studies have generally found mixed results with regards to response inhibition in euthymic participants, however studies with superior methodology have often reported significant results. Hıdıroğlu et al. [61] recruited BD patients that were euthymic for at least 6 months and controlled for other psychiatric comorbidities, finding that BD participants performed worse than healthy controls in the SST. Similarly, Carrus and colleagues [17] showed that euthymic BD individuals performed worse on the HSCT compared to controls, while controlling for sex differences. In sum, difficulties in response inhibition may be relatively stable across illness presentations in BD, representing a trait-like feature of the disorder. However, an alternative explanation may be that these difficulties only emerge after onset of BD and may be accentuated by repeated mood episodes. Indeed, in a cross-sectional study, Wessa and colleagues [69] reported no significant differences in response inhibition performance between individuals at high-risk for BD compared to controls. However, high-risk individuals did show increased BIS scores, as well as poorer delayed gratification abilities on the CGT. Longitudinal studies that measure impulsivity in at-risk individuals before illness onset are required to confirm the nature of impulsivity in BD and its possible prognostic value.

Similarly, inattention was significantly higher in euthymic BD than controls, with a large effect size. However, it is important to note that only a small number of studies were included in this analysis, and all used the same task, the CPT. As previously described, the CPT measures inattention through omission errors (i.e., failing to respond when required). There are many factors which may affect attentional processes, and these are not always controlled for in individual studies. For example, sleep deprivation can negatively impact performance on the CPT [70], while stimulants such as nicotine can improve performance [71]. Further, BD is highly comorbid with other disorders that may impact attentional processes, such as attention deficit hyperactivity disorder (ADHD) [72]. As we wanted to keep our results as generalisable as possible, we did not exclude studies that included BD patients with Axis I comorbidities. This makes it difficult to differentiate whether inattention is related to BD, or other comorbidities. In addition, there is some debate as to whether inattention can be considered a component of impulsivity, or whether they are two distinct psychological processes [68]. It is likely that these are interrelated processes, with difficulties in the attention system resulting in impulsive behaviour. Regardless, neurocognitive dysfunctions are increasingly recognised as a treatment target for improving functioning in BD [73]. Improving cognitive functioning may therefore have downstream effects on impulsive behaviours in BD.

Studies measuring delay of gratification in BD are sparse, and this finding is similar to those from previous reviews. Although no significant differences between euthymic BD and controls were found in the two studies included here, we cannot yet rule out possible differences in delay of gratification in euthymic BD. Indeed, a previous meta-analysis of self-report studies found that scores on the non-planning subscale of the BIS were higher in euthymic BD compared to controls [25]. Similar to other aspects of impulsivity, a host of cognitive processes are involved in delay of gratification, such as reward learning and decision making. Although these areas were outside the scope of the current review, these factors should be considered in future behavioural studies. Indeed, in one of our included studies, euthymic BD participants showed differential reward and punishment learning compared to controls, despite no differences in the delay of gratification task [21]. Specifically, euthymic BD showed a strong negative correlation between reward and punishment learning conditions on the probabilistic classification task (PCT), while there was no such association in controls. This suggests that while BD participants were able to learn either from reward or punishment, they had difficulty alternating between the two tasks. Therefore, it is possible that difficulties in cognitive flexibility may have wider impacts on decision making behaviour.

### Limitations

There are several methodological limitations to the current review. Firstly, as mentioned previously, comorbidities associated with BD may impact impulsivity. Although we excluded studies that explicitly included participants with personality disorders to control for this at least partly, not all studies screened for these comorbidities. Secondly, self-reported impulsivity may be subject to recall bias, whereby euthymic individuals may recall instances when they have been impulsive during periods of mania, rather than euthymic periods. Indeed, the largest effect size in this review was for self-report-measured impulsivity. For this reason, some have argued that behavioural measures are a better measure of trait impulsivity when assessed during euthymia [23]. Nonetheless, medium to large effect sizes were seen in our behavioural meta-analyses, suggesting possible trait characteristics. Thirdly, we did not examine possible medication effects on impulsivity. There is evidence to suggest worse response inhibition and inattention performance in manic BD participants who are taking lithium compared to those who are not [18]. However, a study in euthymic BD participants found no such association [57]. Other medications, such as antipsychotics, are also found to impact cognitive performance, however these effects have rarely been studied in BD populations. On the other hand, including BD populations with diverse medications reflects clinical reality, and taking patients off these medications for study participation would likely raise ethical concerns. Finally, studies often did not report enough information on potential sources of heterogeneity, therefore we did not perform moderator analyses.

## 5. Conclusions

Although impulsivity is a key feature of mania in BD, our results suggest elevated impulsivity remains in euthymic individuals. This was true for self-reported impulsivity, as well as inattention and response inhibition measured via behavioural task performance. Our results suggest impulsivity may be a trait of BD, however they do not rule out the possibility that certain aspects of impulsivity are accentuated during mood episodes. While studies in euthymic BD patients can provide promising insights into possible traits of the disorder, other study designs are required to confirm our findings. Studies assessing impulsivity in individuals at a high-risk of developing BD are particularly promising, as these designs remove the possible impact of historic repeated mood episodes. For example, Kwapil et al. [74] reported higher impulsivity in those at high-risk for BD compared to controls, and those with a higher score were more likely to have a BD diagnosis 13 years later. These findings highlight the possible prognostic value of trait impulsivity, for which preventative or targeted treatment could be delivered. In addition, cognitive interventions that target aspects of impulsivity could provide downstream effects on suicidality and risky behaviour such as substance use, improving the outcomes for those with BD.

## Figures and Tables

**Table 1:** Study characteristics of self-report impulsivity studies.

Study	*N*	Mean Age (SD)	Sex (% Female)	Measure of Impulsivity	Impulsivity Mean (SD)
Baldaçara et al. [2]	BD = 40 HC = 22	40.91 (10.02) 37.72 (13.63)	72.50% 54.54%	BIS-11	64.35 (13.10) 58.52 (9.05)
Cheema et al. [37]	BD = 21 HC = 23	34.8 (11.0) 30.1 (9.2)	38.10% 47.83%	BIS-11	65.7 (10.2) 51.1 (5.8)
Choi et al. [38]	BD = 62 HC = 62	37.0 (10.9) 37.1 (11.0)	56.40% 56.40%	BIS-11	62.00 (11.80) 55.68 (7.73)
Çörekçioğlu et al. [39]	BD = 108 HC = 50	33.80 (9.21) 32.80 (8.37)	48.15% 46.0%	BIS-11	62.66 (10.91) 55.48 (10.11)
Das et al. [32]	BD = 16 HC = 13	35.63 (10.71) 31.15 (11.07)	100% 100%	BIS-11	70.06 (11.12) 59.77 (10.94)
de Almeida et al. [9]	BD = 67 HC = 70	39.6 (9.2) 36.8 (5.6)	64% 70%	BIS-11	69.16 (11.85) 57.13 (6.80)
Ekinci et al. [40]	BD = 71 HC = 50	33.85 (8.75) 31.90 (9.51)	52.1% 52%	BIS-11	74.33 (7.85) 50.36 (3.48)
Eroglu and Lus [33]	BD = 58 HC = 59	31.5 (7.1) 36.0 (5.4)	100% 100%	BIS-11	65.53 (10.28) 63.11 (8.89)
Henna et al. [41]	BD = 54 HC = 136	36.8 (12.1) 37.1 (14.0)	64.8% 64.7%	BIS-11A	73.9 (13.2) 53.2 (9.1)
Hıdıroğlu et al. [42]	BD = 30 HC = 30	35.50 (10.63) 35.73 (10.23)	63.30% 63.30%	BIS-11	59.90 (9.93) 52.90 (7.25)
Ibanez et al. [14]	BD = 13 HC = 25	40.1 (9.4) 35.1(11.2)	38.46% 36%	BIS-11	54.2 (22.3) 40.9 (12.8)
Izci et al. [43,44]	BD = 101 HC = 50	35.69 (12.10) 32.00 (9.24)	29.70% 40%	BIS-11	67.35 (17.85) 55.86 (7.86)
Lewis et al. [36]	BD = 36 HC = 30	50 (10) ^a^ 48.6 (10.5)	73.58% ^a^ NR	BIS-11	58.7 (8.2) 60.8 (10.0)
Lois et al. [45]	BD = 41 HC = 41	44.6 (13.4) 45.3 (13.8)	51.2% 51.2%	BIS-11	64.3 (10.2) 56.2 (7.7)
Lombardo et al. [8]	BD = 54 HC = 49	31.9 (11.0) 30.4 (10.7)	66.7% 57.1%	BIS-11	72.9 (12.1) 52.4 (8.9)
Mazer et al. [35]	BD = 16 HC = 15	37.3 (10.3) 30.8 (7.11)	NR NR	BIS-11	62.15 (21.16) 55.83 (10.30)
Nery-Fernandes et al. [46]; Rocha et al. [47]	BD = 40 HC = 22	40.96 (10.02) 37.7 (13.5)	72.5% 54.55%	BIS-11	62.58 (11.94) 58.5 (9.0)
Ozten and Erol [48]	BD = 78 HC = 70	35.42 (11.15) 37.19 (11.54)	54% 51.5%	BIS-11A	64.37 (9.67) 55.54 (6.87)
Peluso et al. [49]	BD = 12 HC = 51	36.8 (9.7) 34.6 (10.9)	66.7% 66.7%	BIS-11	75.0 (15.1) 56.1 (8.2)
Rote et al. [3]	BD = 97 HC = 50	43.86 (13.82) ^a^ 40.49 (13.56) ^a^	66.67% ^a^ 54.90% ^a^	BIS-11	63.10 (9.49) 58.42 (7.70)
Saunders et al. [34]	BD = 20 HC = 20	36.1 (10.60) 32.7 (10.02)	100% 100%	BIS-11	67.85 (14.31) 55.4 (8.14)
Scholz et al. [50]	BD = 24 HC = 24	44 (10) 44 (10)	41.67% 41.67%	BIS-11	62 (8) 55 (8)
Strasser et al. [51]	BD = 40 HC = 30	48.00 (21.00) 38.50 (21.00)	52.50 % 60.00 %	BIS-11	63.24 (9.76) 58.23 (7.67)
Swann et al. [52]	BD = 22 HC = 35	33 (8)^a^ 35 (10)	51.28% ^a^ 51.43%	BIS-11	77.1 (13.8) 59.9 (9.3)
Swann et al. [53]	BD = 29 HC = 71	36.08 (8.61) ^a^ 32.80 (11.15)	46.43% ^a^ 56.34%	BIS-11	80.2 (13.8) 56.6 (9.4)
Tu et al. [54]	BD = 59 HC = 56	35.5 (8.6) 33.9 (7.6)	52.54% 60.71%	BIS-11	66.61 (9.88) 58.95 (7.47)
Tunc and Kose [55]	BD = 60 HC = 70	33.42 (11.18) 33.43 (10.37)	44.8% 55.2%	BIS-11	68.70 (12.36) 59 (9.39)

SD = standard deviation; BD = bipolar disorder; HC = healthy control; BIS-11 = Barratt Impulsiveness Scale; BIS-11A = Barratt Impulsiveness Scale, version 11A; NR = not reported. ^a^ Demographic characteristics reported for the entire sample; *N* refers to the smaller sub-sample who responded to the impulsivity questionnaire.

**Table 2:** Study characteristics of behavioural impulsivity studies.

Study	*N*	Mean Age (SD)	Sex (% Female)	Impulsivity Paradigm	Impulsivity Domain	Impulsivity Mean (SD)
Akbari et al. [56]	BD = 35 HC = 30	28.80 (2.44) 25.98 (2.76)	48.57% 40%	Continuous Performance Test	Response inhibition	Commission errors BD: 1.60 (1.99) Commission errors HC: 0.09 (0.30)
					Inattention	Omission errors BD: 3.1 (3.7) Omission errors HC: 0.45 (0.52)
Bersani et al. [57]	BD = 30 HC = 15	44.27 (9.09) 42.87 (12.38)	40% 53.34%	Stop Signal Task	Response Inhibition	SSRT (ms) BD: 227.04 (72.30) SSRT (ms) HC: 196.43 (33.30)
Carrus et al. [17]	BD = 86 HC = 46	46.75 (11.19) 42.65 (11.30)	58.14% 54.35%	Hayling Sentence Completion Task	Response Inhibition	Category A errors BD: 8.54 (10.89) Category A errors HC: 2.37 (3.37)
Cheema et al. [37]	BD = 21 HC = 23	34.8 (11.0) 30.1 (9.2)	38.10% 47.83%	Emotional Go/No-Go task	Response Inhibition	Total commission errors BD: 6.7 (7.1) Total commission errors HC: 3.4 (2.6)
Dev et al. [58]	BD = 28 HC = 36	46.97 (11.43) 49.93 (12.39)	75% 67%	D-KEFS Colour Word Interference	Response Inhibition	Inhibition scaled score BD: 10.04 (3.12) Inhibition scaled score HC: 11.40 (2.4)
Duek et al. [21]	BD = 40 HC = 41	42.15 (11.94) 38.90 (11.19)	45% 43.90%	Single Key Impulsivity Paradigm	Delay of Gratification	Total number of presses BD: 785 (13.91) Total number of presses HC: 671 (11.40)
Farahmand et al. [59]	BD = 30 HC = 29	32.6 (7.85) 32.43 (7.64) ^a^	50% 50%^a^	Stop Signal Task	Response Inhibition	SSRT BD: 368.22 (163.65) SSRT HC: 216.65 (111.75)
Feliu-Soler et al. [60]	BD = 35 HC = 70	39.57 (8.27) 36.59 (8.82)	57.15% 68.57%	Continuous Performance Test II	Response Inhibition	Commissions BD: 13.24 (8.82) Commissions HC: 8.77 (6.71)
					Inattention	Omissions BD: 5.71 (7.35) Omissions HC: 1.29 (3.15)
Frangou et al. [13]	BD = 10 HC = 43	53 (8.3) 42.9 (11.2)	70% 44.19%	Hayling Sentence Completion Task	Response Inhibition	Category A errors BD: 6.60 (0.84) Category A errors HC: 4.77 (1.85)
Hıdıroğlu et al. [61]	BD = 35 HC = 33	34.4 (7.8) 32.3 (8.5)	60% 63.6%	Stop Signal Task	Response Inhibition	SSRT (ms) BD: 313.48 (58.47) SSRT (ms) HC: 256.32 (46.68)
Ibanez et al. [14]	BD = 13 HC = 25	40.1 (9.4) 35.1 (11.2)	38.46% 36%	Go/No-go task	Response Inhibition	Commission errors (%) BD: 7.6 (19.8) Commission errors (%) HC: 0.37 (2.0)
Kaladjian et al. [7]	BD = 10 HC = 10	40.1 (13.7) 41.5 (13.4)	50% 50%	Go /No-go task	Response Inhibition	Commission errors (%) BD: 9.00 (5.83) Commission errors (%) HC: 6.00 (7.48)
Kaladjian et al. [62]	BD = 20 HC = 20	37.9 (11.4) 34.6 (10.6)	50% 50%	Go/ No-go task	Response Inhibition	Commission errors (%) BD: 7.3 (5.5) Commission errors (%) HC: 8.9 (7.2)
Kollmann et al. [22]	BD = 54 HC = 54	42.63 (9.18) 43.07 (9.21)	46.3% 46.3%	Stop Signal Task	Response Inhibition	SSRT (ms) BD: 197.60 (62.05) SSRT (ms) HC: 180.11 (41.27)
				Cambridge Gambling Task	Delay of Gratification	Delay aversion BD: 0.23 (0.17) Delay aversion HC: 0.21 (0.18)
Kolur et al. [19]	BD = 30 HC = 30	22.40 (2.52) 22.50 (2.32)	30% 30%	Continuous Performance Test	Response Inhibition	Total commission errors BD: 21.40 (29.91) Total commission errors HC: 12.73 (7.15)
					Inattention	Total omission errors BD: 17.33 (8.74) Total omission errors HC: 7.80 (4.79)
Malloy-Diniz et al. [63]	BD = 95 HC = 94	41 (12) 32 (13)	69.5% 56.4%	Continuous Performance Test II	Response Inhibition	Commission errors BD: 16.17 (8.76) Commission errors HC: 10.26 (7.20)
					Inattention	Omission errors BD: 9.31 (15.68) Omission errors HC: 3.47 (5.38)
Morsel et al. [64]	BD = 20 HC = 18	44 (12.05) 42 (15.09)	50% 60%	Go/No-go task	Response Inhibition	Commission errors (%) BD: 9.81% (9.92) Commission errors (%) HC: 6.26% (6.17)
Okasha et al. [4]	BD = 60 HC = 30	27.02 (5.70) 25.77 (3.88)	66.7% 33.3%	Continuous Performance Test	Response Inhibition	Total commission errors BD: 9.03 (8.025) Total commission errors HC: 4.20 (0.997)
					Inattention	Total omission errors BD: 10.22 (12.013) Total omission errors HC: 4.20 (1.627)
Robinson et al. [65]	BD = 22 HC = 21	43.14 (7.8) 43.57 (6.5)	63.64% 52.38%	Modified Continuous Performance Test	Response Inhibition	d’ BD: 3.26 (0.92) d’ HC: 3.87 (0.81)
Sarnicola et al. [66]	BD = 71 HC = 82	43.8 (11.4) 40.5 (11.6)	53.52% 52.44%	Hayling Sentence Completion Task	Response inhibition	Category A error BD: 2.50 (2.57) Category A error HC: 1.09 (2.32)
Swann et al. [52]	BD = 25 HC = 35	33 (8) ^a^ 35 (10)	51.28%^a^ 51.43%	Immediate Memory Task	Response Inhibition	Commission error rate (%) BD: 20.4 (10.2) Commission error rate (%) HC: 17.6 (14.1)
Swann et al. [67]	BD = 30 HC = 37	37.53 (10.87) 35.63 (8.23)	43.34% 54.05%	Immediate Memory Task	Response Inhibition	Commission errors (%) BD: 22.99 (11.49) Commission errors (%) HC: 17.8 (13.7)

SD = standard deviation; BD = bipolar disorder; HC = healthy control. ^a^ Demographic characteristics reported for the entire sample; *N* refers to the smaller sub-sample who completed the impulsivity task.

## Data Availability

Not applicable.

## Supplementary Materials

The following supporting information can be downloaded at: https://www.mdpi.com/article/10.3390/brainsci12101351/s1, Table S1: Quality assessment of included studies.
