# Supplementary material for: Impulsivity in Bipolar Disorder: State or Trait?
Source: Brain Sci. 2022 Oct 5;12(10):1351. doi: 10.3390/brainsci12101351 (PMC9599710; doi:10.3390/brainsci12101351)
Supplement: Supplementary file 1 [file brainsci-12-01351-s001.zip › Supplementary Table S1.pdf]

Table S1. Quality assessment of included studies.

Study	Quality assessment item 1	2	3	4	5	6	7	8	9	10	11	Overall summary score
Akbari et al. [56]	1	1	1	2	1	0	2	1	1	1	1	0.55
Baldaçara et al. [2]	2	2	1	2	2	0	2	2	1	2	2	0.82
Bersani et al. [57]	2	1	1	2	2	0	2	2	1	2	2	0.77
Carrus et al. [17]	2	2	1	2	2	1	2	2	1	2	1	0.82
Cheema et al. [37]	2	2	2	2	2	1	2	2	2	2	2	0.95
Choi et al. [38]	1	1	1	2	2	1	2	2	1	2	2	0.77
Çörekçioğlu et al. [39]	1	1	1	2	2	1	2	2	1	2	2	0.77
Das et al. [32]	2	2	1	2	1	0	2	2	1	2	1	0.73
de Almeida et al. [9]	2	2	1	1	1	1	2	2	1	2	2	0.77
Dev et al. [58]	2	2	1	2	1	1	2	2	2	2	2	0.86
Duek et al. [21]	2	2	1	2	1	1	2	2	1	2	1	0.77
Ekinci et al. [40]	1	2	2	2	2	1	2	1	2	2	2	0.86
Eroglu & Lus [33]	2	2	1	2	1	1	1	2	1	2	1	0.73
Farahmand et al. [59]	2	2	1	2	2	1	2	2	1	2	2	0.86
Feliu-Soler et al. [60]	1	2	1	1	2	1	2	2	1	2	2	0.77
Frangou et al. [13]	2	2	1	2	2	0	1	2	1	2	1	0.73
Henna et al. [41]	2	2	1	2	2	1	2	2	0	2	2	0.82
Hıdıroğlu et al. [42]	2	2	1	2	2	1	2	2	1	2	2	0.86
Hidiroğlu et al. [61]	2	2	1	2	2	1	2	2	1	2	2	0.86
Ibanez et al. [14]	2	2	1	2	1	0	2	2	1	2	1	0.73
Izci et al. [43]	1	2	1	2	2	1	1	2	1	2	2	0.77
Izci et al. [44]	1	2	1	2	2	1	2	2	1	2	1	0.77
Kaladjian et al. [7]	2	2	1	2	2	0	1	2	1	2	1	0.73
Kaladjian et al. [62]	2	2	1	2	2	1	2	2	1	2	2	0.86
Kollmann et al. [22]	2	2	1	2	2	0	2	2	1	2	2	0.82
Kolur et al. [19]	1	2	2	2	1	1	2	2	2	2	2	0.86
Lewis et al. [36]	2	2	1	1	1	1	1	2	2	2	2	0.77
Lois et al. [45]	2	2	1	2	1	1	2	2	2	2	2	0.86
Lombardo et al. [8]	2	2	2	2	1	1	2	2	1	2	2	0.86
Malloy-Diniz et al. [63]	1	2	2	2	2	1	2	2	2	2	2	0.91
Mazer et al. [35]	1	2	1	2	1	1	2	2	1	2	2	0.77
Morsel et al. [64]	2	2	1	2	2	1	2	2	1	2	1	0.82
Nery-Fernandes et al. [46]	1	1	1	2	1	1	2	1	1	2	2	0.68
Okasha et al. [4]	1	2	1	2	1	1	1	2	1	2	2	0.73
Ozten & Erol [48]	1	2	1	2	2	1	1	2	0	1	2	0.68
Peluso et al. [49]	1	2	1	2	1	0	2	2	1	2	2	0.73
Robinson et al. [65]	2	2	2	2	2	1	2	2	2	2	1	0.91
Rocha et al. [47]	1	2	1	2	2	1	2	2	1	2	2	0.82
Rote et al. [3]	2	2	2	2	2	1	2	2	2	2	2	0.95
Sarnicola et al. [66]	2	0	1	2	2	1	2	2	1	2	2	0.77

Saunders et al. [34]	2	2	1	1	2	1	2	2	1	2	2	0.82
Scholz et al. [50]	1	1	2	2	2	1	2	2	2	2	2	0.86
Strasser et al. [51]	2	2	1	2	2	1	2	2	2	2	2	0.91
Swann et al. [52]	1	2	1	1	2	1	0	1	0	2	1	0.55
Swann et al. [67]	2	1	1	1	2	1	1	2	2	2	2	0.77
Swann et al. [53]	1	1	1	2	2	0	2	2	1	2	2	0.73
Tu et al. [54]	2	1	1	2	2	1	2	2	2	2	2	0.86
Tunc & Kose [55]	1	2	1	1	2	1	2	2	0	1	1	0.64

Item numbers correspond to the domains delineated below. Items are rated as 2 = 'yes'; 1 = 'partial'' or 0 = "no". Summary scores are calculated as the sum of all ratings divided by the total possible score. Summary scores closer to 1 indicate higher quality.

Quality assessment items: 1. Question/objective sufficiently described; **2.** Study design evident and appropriate; **3.** Method of subject/comparison group selection or source information/input variables described and appropriate; **4.** Subject and comparison group characteristics sufficiently described; **5.** Outcome measure(s) well defined and robust to measurement/misclassification bias; means of assessment reported; **6.** Sample size appropriate; **7.** Analytic methods described/justified and appropriate; **8.** Some estimate of variance is reported for the main results; **9.** Controlled for confounding; **10.** Results reported in sufficient detail; **11.** Conclusions supported by the results.
